# Expression of miRNAs in ovine fetal gonads: potential role in gonadal differentiation

**DOI:** 10.1186/1477-7827-9-2

**Published:** 2011-01-11

**Authors:** Katie J Torley, Juliano C da Silveira, Peter Smith, Russell V Anthony, DN Rao Veeramachaneni, Quinton A Winger, Gerrit J Bouma

**Affiliations:** 1 Animal Reproduction and Biotechnology Laboratory, Department of Biomedical Sciences, Colorado State University, Fort Collins, CO 80523, USA; 2 AgResearch Limited, Invermay, Research Centre Mosgiel, New Zealand

## Abstract

**Background:**

Gonadal differentiation in the mammalian fetus involves a complex dose-dependent genetic network. Initiation and progression of fetal ovarian and testicular pathways are accompanied by dynamic expression patterns of thousands of genes. We postulate these expression patterns are regulated by small non-coding RNAs called microRNAs (miRNAs). The aim of this study was to identify the expression of miRNAs in mammalian fetal gonads using sheep as a model.

**Methods:**

We determined the expression of 128 miRNAs by real time PCR in early-gestational (gestational day (GD) 42) and mid-gestational (GD75) sheep ovaries and testes. Expression data were further examined and validated by bioinformatic analysis.

**Results:**

Expression analysis revealed significant differences between ovaries and testes among 24 miRNAs at GD42, and 43 miRNAs at GD75. Bioinformatic analysis revealed that a number of differentially expressed miRNAs are predicted to target genes known to be important in mammalian gonadal development, including *ESR1, CYP19A1*, and *SOX9*. In situ hybridization revealed *miR-22 *localization within fetal testicular cords. As estrogen signaling is important in human and sheep ovarian development, these data indicate that miR-22 is involved in repressing estrogen signaling within fetal testes.

**Conclusions:**

Based on our results we postulate that gene expression networks underlying fetal gonadal development are regulated by miRNAs.

## Background

Genetic sex in mammals is determined at the time of fertilization; fertilization of eggs with X or Y-bearing sperm will yield XX (female) or XY (male) embryos, respectively. Normally, distinct genetic pathways will subsequently direct undifferentiated genital ridges in XX and XY fetuses to develop into fetal ovaries or fetal testes, respectively. Mammalian fetal gonadal differentiation is a developmental process involving a dose dependent balance between promoting and antagonizing factors. That is, the testicular developmental pathway involves genetic networks both promoting testis development and preventing ovarian development and vice versa [1,2]. Critical genes involved in initiation of the testicular and ovarian developmental pathways are the Y-linked gene, *Sry *(sex determining region of chromosome Y) [3,4], and *Rspo1 *(R-spondin homolog), *Wnt4 *(wingless-related MMTV integration site 4) and β-catenin [5,6], respectively. These genes are expressed in the somatic support cells of the fetal gonads directing differentiation of the supporting cell lineages, i.e., Sertoli cells in the testis and granulosa cells in the ovary [5-9]. Genome profiling experiments further have demonstrated that both testicular and ovarian developmental pathways are characterized by dynamic expression patterns of thousands of genes [10-14]. How the expression and function of these genes are regulated is unknown.

Small non-coding RNA molecules called microRNAs (miRNAs) are ~22 nt cytoplasmic RNAs that regulate gene expression and function in many tissues [15-17]. MiRNAs are transcribed by RNA polymerase II generating hairpin loop containing structures called primary-miRNAs which are cleaved by the endonuclease RNAse III DROSHA and its partner DGCR8, yielding a 70-90 nt hairpin stem-loop precursor miRNA (pre-miRNA). Pre-miRNAs are exported into the cytoplasm by Exportin-5 and processed by DICER1, yielding a ~22 nt mature miRNA. MiRNAs recognize transcript targets through base-pairing to the 3'-untranslated region (UTR), and are able to repress translation or cause degradation, depending upon sequence complementarity. Previous work has demonstrated that miRNA sequences are highly conserved across species and can be expressed in a tissue specific manner [18]. The importance of miRNAs in reproduction was demonstrated using transgenic mouse models [19-24]. Conditional gene targeting approaches demonstrated that Dicer is important for primordial germ cell and spermatogonial proliferation [19,20], Sertoli cell function [21], and development of the oviducts and uteri [22-24].

The above-mentioned studies indicate that miRNAs are important for reproductive development and function, but do not indicate which miRNAs. MiRNA cloning experiments have demonstrated differences in expression profiles between ovaries and testis of adult mice [25], and analysis of a bovine fetal ovarian miRNA library revealed miRNAs predominantly expressed in fetal ovaries compared to somatic tissue pools [26]. Little is known about the expression of miRNAs during fetal gonadal development in mammals. Based on the observation that fetal ovarian and testicular development involves coordinate expression of thousands of genes, we predict that miRNAs are expressed and are involved in regulating gene expression and function during fetal gonadal development.

The aim of this study was to identify the expression of miRNAs in mammalian fetal gonads using the ovine as a model. In addition, expression levels were examined of a number of key genes involved in fetal ovarian (*ESR1, ESR2, CYP19, FST*, and *WNT4*) and testicular development (*SOX9*) as potential target genes of miRNAs. The study of gonadal differentiation and reproductive development in sheep provides both insight into human gonadal development, and a better understanding of reproductive development of economically important livestock species.

## Methods

### Sheep breeding and tissue collection

All experimental procedures using animals were approved by the Colorado State University Animal Care and Use Committee. Estrous cycles of twenty-four ewes were synchronized using prostaglandin F2α (5 mg; Lutalyse; Pfizer Animal Health), and ewes and rams were mated and kept together for twelve to twenty-four hours to ensure successful mating (up to 4 ewes with one ram, and the ram replaced with a new ram after ~12 hours). Around gestational day (GD) 40, pregnancy status was confirmed by ultrasonography.

Tissues were collected at either GD42 or 75 (term is ~150 days). GD42 corresponds to the period of testicular cord differentiation and ovigerous cord development in XY and XX gonads respectively, whereas GD75 coincides with primordial follicle formation in the ovary [27,28]. Ewes were taken off feed and water at least twelve hours before necropsy to facilitate unhindered tissue collection. Ewes were euthanized by intrajugular injection of sodium pentobarbital (90 mg/kg). Each fetus was measured crown to rump and lengths were used to estimate and confirm gestational age. Fetal gonads were removed and one was homogenized in 350 μl of RLT Plus lysis buffer (RNeasy Plus Mini Kit, Qiagen) and the other was fixed in 4% paraformaldehyde (PFA; GD42) or Bouin's fixative (GD75). Gonads used for RNA isolation were stored at -80°C. Gonads for histology were fixed overnight, transferred to 70% ethanol, and stored at 4°C until embedded in paraffin.

### Fetal sheep sex genotyping

Genetic sex was determined by PCR genotyping using fetal tail tissue lysate, and ovine *SRY *primers (Forward primer: 5'- CATTGTGTGGTCTCGTGAACG-3'; Reverse primer: 5'-GTCTCGGTGTATAGCTAGTAG-3') designed based on the ovine SRY sequence (GenBank Accession number Z30265). Polymerase chain reaction was run using the following program: 95°C - 3 minutes; 60°C - 5 minutes; 72°C - 5 minutes (1 cycle); 95°C - 30 seconds, 58°C - 30 seconds, 72°C - 45 seconds (35 cycles); and 72°C - 5 minutes. PCR products were run on a 2% agarose gel, and visualized using ethidium bromide.

### Total RNA isolation

Total RNA, including miRNAs, was isolated using the RNeasy Plus Mini kit (Qiagen), according to the manufacturer's instructions. To ensure the small RNA fraction was retained, the first washing step with RW1 buffer was replaced by 100% ethanol (1.5× volume of the tissue lysate) according to manufacturer's specification. RNA was eluted in 40 μl RNAse-free water, and treated with DNAse (4 μl 10× DNAse buffer and 1 μl DNAse-I (Ambion)) to eliminate genomic DNA contamination. RNA concentration and purity were determined using the NanoDrop ND-1000 spectrophotometer. Samples were then stored at -80°C.

### Reverse Transcription of miRNAs

Small non-coding RNAs were reverse transcribed using the QuantiMirTM RT kit (Systems Biosciences (SBI), Mountain View, CA) according to the manufacturer's instructions. Briefly, ~500 ng of total RNA including the small RNA fraction was anchor-tailed with polyA by incubating RNA, 5× PolyA Buffer, 25 mM MnCl2, 5 mM ATP, and polyA polymerase at 37°C for 30 minutes. Oligo dT adaptors were annealed at 60°C for 5 minutes, and reverse transcription first strand synthesis reaction was carried out by incubating the samples at 42°C for 60 minutes followed by 95°C for 10 minutes.

### Real-time PCR expression of conserved miRNAs in fetal gonads

A preliminary experiment was conducted to examine the expression of 211 miRNAs whose mature sequence was identical in human, mouse, bovine and/or goat [29]. Of these 211 miRNAs, 128 were selected that were expressed (arbitrarily set at crossing-point value (Cp) < 37) according to real time RT-PCR analysis, and showed a single melt peak following dissociation curve analysis. The relative expression of 128 mature miRNAs (see Additional file 1, Table S1) in fetal sheep gonads was assessed using real-time PCR using a custom designed primer plate containing the mature miRNA sequences as a forward primer (SBI).

Each analysis was performed in 6 μl reactions containing 2× SYBR Green I master mix (Roche Applied Sciences), 10 μM Universal reverse primer and miRNA specific forward primer (SBI), and 1 μl cDNA. Real time PCR was conducted using the LightCycler480 PCR system (Roche Applied Sciences) with 384-well plates each containing 3 biological replicates. The PCR cycle conditions were as follows: 95°C for 5 minutes, 45 cycles of 95°C for 10 seconds, 60°C for 15 seconds, and 72°C for 15 seconds followed by a melt curve analysis to confirm amplification of single cDNA products. Fold change and statistically significant differences of the 128 miRNAs were determined using Global Pattern Recognition (GPR) software v2.0 [30,31]. Using GPR, miRNAs considered significantly different were those with a GPR score of 0.400 or greater [31], whereas fold changes were calculated based on 10 normalizers (miRNAs in the data set that are expressed and not significantly different).

### Reverse transcription of mRNAs

Messenger RNA was reverse transcribed into cDNA using the MessageSensorTM RT kit (Ambion Inc.) as described previously [31]. Briefly, 5 μl of RNA (50 ng/μl) was combined with 10× RT buffer, dNTPs, 10 μM random decamers, RNase Inhibitor, and M-MLV reverse transcriptase. RNA was reverse transcribed by incubating the samples at 25°C for 10 minutes, 42°C for 60 minutes, and 95°C for 10 minutes. cDNA was used immediately for real time PCR analysis.

### Real-time PCR expression of mRNAs in fetal gonads

The relative expression level of mRNAs involved in fetal gonadal development (*ESR1, ESR2, CYP19, SOX9, WNT4, FST*) and two housekeeping genes (*GAPDH *and *RN18S*) was examined using real-time PCR. Preliminary experiments in our laboratory revealed the expression level of these two housekeeping genes were consistent and did not change. Gene specific primers were designed using Primer3 [32] using ovine, bovine, and/or porcine sequences (see Additional file 2, Table S2). Amplification efficiencies were determined using a 10 fold serial dilution series of a GD75 XX and XY gonadal cDNA pool for each primer set. In addition, cDNA products were sequenced to validate primer specificity.

Analysis was performed in 10 μl reactions containing 2× SYBR Green Master Mix I (Roche Applied Sciences), 0.5 μM gene specific forward and reverse primer, and cDNA (diluted 1:4) using the LightCycler480 PCR system. The reaction conditions were as follows: 95°C for 5 minutes and then 45 cycles of 95°C for 10 seconds, 60°C for 30 seconds, and 72°C for 30 seconds followed by a melt curve analysis, to confirm amplification of single PCR products. This experiment was repeated twice (n = 2-4). Relative expression level of transcripts was determined by calculating the geometric mean of *GAPDH *and *RN18S *expression values, and using this as a normalization factor. Statistical differences were assessed at P < 0.05 using a Students t-test. PCR amplification efficiencies were between 1.8 - 2.1, and relative expression levels were presented by plotting mean 2^-ΔCp ^values [33].

### Histology

Fixed fetal gonads were dehydrated and paraffin embedded using routine procedures. 5 μm-thick tissue sections were cut and stained with hematoxylin and eosin, and examined using a light microscope equipped with plan apochromatic objectives. Additional 4% paraformaldehyde fixed, unstained 5 μm GD75 and GD90 tissue sections were provided by Dr. Peter Smith (AgResearch Limited, Invermay, New Zealand) and used for in situ hybridization analysis.

### In situ hybridization

Cellular localization of miR-22 was examined in GD75 and GD90 ovaries and testes using non-radioactive in situ hybridization. Non-radioactive in situ hybridization was performed using DIG labeled LNA miRNA probes for *U6 *(positive control), *miR-22*, and a scramble (negative control) (Cat # 99002-15, 300500-15, and 99004-01 respectively; Exiqon, Vedbaek, Denmark) according to the manufacturer's instruction and included a Tyramide Signal Amplification (TSA) step (Perkin-Elmer, Waltham, MA). Briefly, following deparaffinization with xylene, tissue sections were rehydrated through a graded series of ethanol washes (100%, 70%, 50%, 25%), and rinsed in phosphate buffered saline (PBS). After endogenous peroxidase activity was blocked by incubating tissue sections in 0.03% H_2_O_2 _(30 minutes) and proteins were digested with 10 μg/ml proteinase K, tissue sections were washed and post-fixed in 4% PFA, and incubated overnight in hybridization buffer (50% deionized formamide, 5× SCC, 0.1% Tween 20, 50 μg/ml heparin, and 50 μg/ml yeast tRNA). The next day, tissue sections were incubated overnight with hybridization buffer containing 0.25 μM probe at 50°C in a humidified chamber. Following washes and incubation in blocking buffer (2% sheep serum and 2 mg/ml BSA in PBS plus 0.01% Tween (PBST)), tissue sections were incubated in blocking buffer containing anti-DIG conjugated horseradish peroxidase (HRP) antibody (Abcam, Cambridge, MA; 1:2000 dilution) for 30 minutes at room temperature. TSA signal amplification was conducted by adding 100-300 μl fluophore tyramide solution per tissue section for 12 minutes at room temperature, followed by washes in PBST, and incubation with anti-fluorescein alkaline phosphatase (AP) conjugated antibody (Rockland Inc., Gilbertsville, PA; 1:75 dilution) for 30 minutes. Antibody staining was visualized using BCIP/NBT in AP buffer (100 mM Tris-HCl, 50 mM MgCl2, 100 mM NaCl, 0.1% Tween 20, 2.4 mg Levamisole (Honeywell, Seelze, Germany)). Color development was monitored and stopped by washing the slides in PBST (5 hours for *U6*, 48 hours for *miR-22 *and scramble).

## Results

### MiRNA expression in fetal gonads

**Figure 1:**
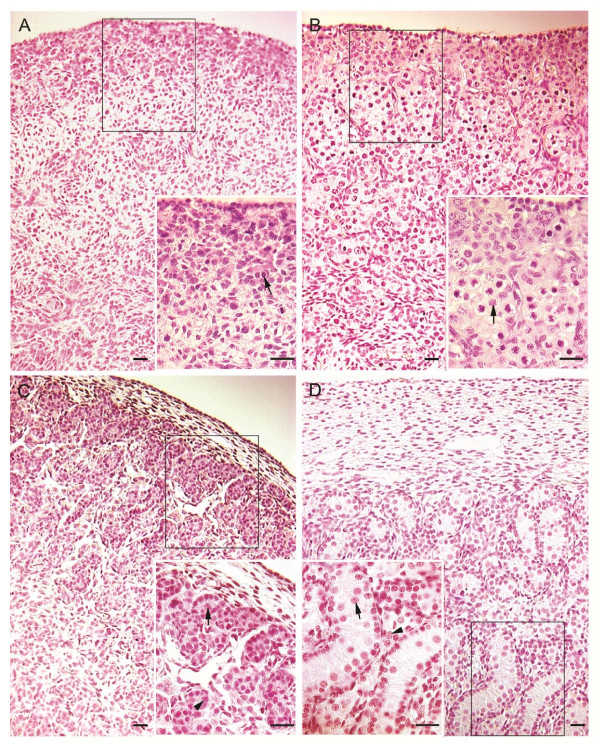
**Tissue sections of GD42 and GD75 ovaries and testes stained with hematoxylin and eosin**. A) GD42 ovary illustrating a thin ovarian cortex containing oogonia and precursor granulosa cells. Arrow in insert points to an oogonium. B) GD75 ovary illustrating a thickened cortex containing ovigerous cords. Arrow in insert depicts oocyte within an ovigerous cord. C) GD42 testis demonstrating presence of testicular cords containing gonocytes and Sertoli cells. Arrow in insert points to a gonocyte; arrowhead points to a Sertoli cell nucleus. D) GD 75 testis revealing prominent testicular cords. Arrow in insert points to a gonocyte; arrowhead points to an interstitial (Leydig) cell. All bars are 20 μm.

Using a custom designed 128-miRNA profiler PCR plate, the relative expression level of 128 conserved miRNA sequences was assessed in sheep fetal gonads. At GD42, when testicular cords develop in XY gonads (Figure 1), 24 miRNAs exhibited a sexual dimorphic expression pattern with at least 2 fold difference (Table 1). Of these, 12 miRNAs were expressed significantly higher in XX and 12 were expressed significantly higher in XY gonads. At GD75, when the ovary is filled with ovigerous cords (Figure 1) and primordial follicles start to form, 43 miRNAs exhibited a sexually dimorphic expression pattern with at least a 2 fold change; 26 miRNAs were expressed significantly higher in GD75 ovaries, and 17 were expressed significantly higher in GD75 testes (Table 2).

**Table 1:** Significant differently expressed miRNAs exhibiting a fold change > 2 in GD42 sheep ovaries and testes, according to real time RT-PCR analysis.

miRNA	GPR Score	Fold-Change
**Significantly higher in GD42 ovary**		
*miR-431*	0.836	5.35
*let-7c*	0.789	3.98
*miR-328*	0.688	3.85
*miR-195*	0.734	3.59
*miR-486*	0.695	3.40
*let-7d*	0.727	3.13
*miR-7*	0.594	3.07
*miR-484*	0.672	2.99
*miR-423*	0.656	2.81
*let-7a*	0.703	2.75
*miR-320*	0.531	2.67
*let-7e*	0.586	2.23
**Significantly higher in GD42 testis**		
*miR-758*	0.734	5.00
*miR-192*	0.844	4.50
*miR-223*	0.797	3.74
*miR-101*	0.477	2.74
*miR-411*	0.656	2.58
*miR-369-5p*	0.523	2.55
*miR-301*	0.594	2.21
*miR-142-5p*	0.484	2.19
*miR-27b*	0.516	2.19
*miR-379*	0.555	2.18
*miR-142-3p*	0.484	2.08
*miR-376a*	0.414	2.04

Significance (indicated by GPR score) and fold change were determined using Global Pattern Recognition v2.0.

**Table 2:** Significant differently expressed miRNAs exhibiting a fold change > 2 in GD75 sheep ovaries and testes, according to real time RT-PCR analysis.

miRNA	GPR Score	Fold-Change
**Significantly higher in GD75 ovary**		
*let-7c*	0.953	100.07
*miR-146b*	0.781	18.52
*miR-103*	0.844	17.23
*miR-125b*	0.859	15.92
*let-7d*	0.758	15.73
*miR-484*	0.859	15.55
*let-7a*	0.758	14.90
*miR-25*	0.773	10.89
*miR-125a*	0.758	8.91
*miR-99a*	0.805	8.78
*miR-100*	0.828	8.39
*miR-150*	0.664	6.25
*miR-130a*	0.734	5.76
*miR-19b*	0.664	5.33
*miR-28*	0.695	4.81
*miR-362*	0.602	4.68
*miR-16*	0.484	4.35
*miR-200c*	0.688	4.16
*miR-200b*	0.688	4.11
*miR-210*	0.688	3.85
*miR-19a*	0.555	3.59
*miR-10a*	0.648	3.12
*let-7g*	0.602	2.82
*let-7e*	0.469	2.48
*miR-92*	0.430	2.42
*miR-183*	0.422	2.42
**Significantly higher in GD75 testis**		
*miR-22*	0.961	4223.97
*miR-142-3p*	0.922	23.40
*miR-27a*	0.734	7.77
*miR-33*	0.719	6.04
*miR-302d*	0.727	5.24
*miR-27b*	0.672	4.65
*miR-410*	0.508	3.81
*miR-199b*	0.492	3.78
*miR-455*	0.570	3.27
*miR-211*	0.547	3.22
*miR-212*	0.422	3.15
*miR-379*	0.523	3.02
*miR-411*	0.453	2.86
*miR-369-5p*	0.531	2.67
*miR-301*	0.453	2.63
*miR-409-5p*	0.445	2.30
*miR-152*	0.484	2.03

Significance (indicated by GPR score) and fold change were determined using Global Pattern Recognition v2.0.

In addition to identifying miRNAs exhibiting sexual dimorphic expression patterns, relative expression level of miRNAs was examined during development within fetal ovaries and fetal testes by comparing miRNA expression in GD42 and GD75 gonads. Comparing miRNA expression in GD42 and GD75 ovaries, 62 miRNAs with at least a 2 fold change were differentially expressed; 31 were expressed significantly higher in GD42 ovaries and 31 were expressed significantly higher in GD75 ovaries (Table 3). Within fetal testes, 30 miRNAs with at least a 2 fold change were expressed differentially when comparing GD42 to GD75; 13 were expressed significantly higher in GD42 testes and 17 were expressed significantly higher in GD75 testes (Table 3).

**Table 3:** Significant differently expressed miRNAs exhibiting a fold change ≥ 2 in GD42 and GD75 sheep ovaries and testes, according to real time RT-PCR analysis.

miRNA	GPR Score	Fold-Change	miRNA	GPR Score	Fold-Change	miRNA	GPR Score	Fold-Change
**Significantly higher in GD42 versus GD75 XX gonads**			**Significantly higher in GD75 versus GD42 XX gonads**			**Significantly higher in GD42 versus GD75 XY gonads**		
*miR-22*	0.953	1935.53	*let-7c*	0.883	46.01	*miR-302d*	0.891	16.36
*miR-302d*	0.945	119.16	*miR-142-5p*	0.898	35.40	*miR-200c*	0.867	11.85
*miR-206*	0.875	14.76	*miR-19b*	0.906	27.28	*miR-222*	0.734	5.32
*miR-222*	0.875	8.43	*miR-19a*	0.883	16.43	*miR-200b*	0.695	4.44
*miR-196a*	0.844	7.40	*miR-135a*	0.781	12.28	*miR-362*	0.609	3.95
*miR-328*	0.672	7.34	*miR-125b*	0.883	12.18	*miR-99b*	0.539	3.95
*miR-433*	0.781	7.23	*let-7a*	0.727	10.89	*miR-485-5p*	0.578	3.61
*miR-486*	0.813	6.81	*miR-130a*	0.852	9.93	*miR-382*	0.586	3.22
*miR-216*	0.727	6.63	*miR-146b*	0.648	9.92	*miR-149*	0.484	3.01
*miR-221*	0.820	5.88	*miR-100*	0.883	9.48	*miR-15b*	0.523	2.82
*miR-196b*	0.758	5.69	*miR-99a*	0.859	8.91	*miR-92*	0.430	2.72
*miR-574*	0.750	4.83	*let-7d*	0.453	8.76	*miR-17-5p*	0.484	2.27
*miR-485-5p*	0.742	4.52	*miR-101*	0.688	8.52	*miR-107*	0.414	2.15
*miR-7*	0.727	4.45	*miR-484*	0.750	5.00	**Significantly higher in GD75 versus GD42 XY gonads**		
*miR-423*	0.617	4.39	*miR-150*	0.750	4.66	*miR-142-3p*	0.938	15.89
*miR-382*	0.680	3.76	*miR-30b*	0.758	4.41	*miR-142-5p*	0.938	14.89
*miR-134*	0.656	3.57	*miR-103*	0.664	4.32	*miR-33*	0.922	11.50
*miR-181b*	0.703	3.54	*let-7g*	0.664	3.94	*miR-211*	0.758	6.22
*miR-200c*	0.625	2.99	*miR-497*	0.773	3.82	*miR-193a*	0.633	4.21
*miR-668*	0.648	2.96	*miR-25*	0.688	3.71	*miR-19b*	0.586	3.59
*miR-149*	0.586	2.90	*miR-331*	0.664	3.59	*miR-19a*	0.602	3.53
*miR-379*	0.672	2.90	*miR-143*	0.734	3.36	*miR-199b*	0.539	3.52
*miR-598*	0.656	2.55	*miR-199a*	0.695	3.18	*miR-27a*	0.406	3.35
*miR-615*	0.430	2.54	*miR-33*	0.656	2.99	*miR-199a*	0.414	3.22
*miR-214*	0.500	2.50	*miR-335*	0.680	2.89	*miR-143*	0.453	3.18
*miR-539*	0.656	2.40	*miR-21*	0.680	2.84	*let-7g*	0.563	2.79
*miR-652*	0.453	2.35	*miR-15a*	0.648	2.76	*miR-22*	0.500	2.62
*miR-17-5p*	0.578	2.31	*miR-148a*	0.641	2.31	*miR-30e-5p*	0.500	2.46
*miR-15b*	0.641	2.27	*let-7e*	0.492	2.31	*miR-204*	0.414	2.27
*miR-296*	0.602	2.21	*miR-204*	0.563	2.11	*miR-30b*	0.492	2.25
*miR-212*	0.539	2.01	*miR-148b*	0.633	2.08	*let-7e*	0.430	2.16

Significance (indicated by GPR score) and fold change were determined using Global Pattern Recognition v2.0.

To gain further insight into the possible function of these miRNAs, TargetScan 5.1, Meta Mir:Target Inference, and miRGator [34-36] were used as tools to identify potential genes known to be involved in mammalian fetal gonadal differentiation targeted by miRNAs. Examining the differentially expressed miRNAs in GD42 gonads revealed a number of genes, including *SOX9, ESR1 *(estrogen receptor 1), and *CYP19A1 *(cytochrome P450, family 19, subfamily a, polypeptide 1) as potential targets (Table 4). Furthermore, differently expressed miRNAs in GD75 ovaries and testes are predicted to target a number of genes including *ESR1*, *CYP19A1, FST *(follistatin), and *WNT4 *(Table 4).

**Table 4:** Selected miRNAs and their predicted gene targets according to TargetScan 5.1 [34] and Meta Mir:Target Inference (MAMI) [35]

Predicted Gene Target	miRNA
*CYP19*	*Let7 (a, c, d, e, g)*
*ESR1*	*miR-22*
*FOG2 (ZFPM2)*	*miR-200c, miR-142-5p, miR-302d*
*FOXL2*	*miR-302d*
*FST*	*miR-410*
*GATA4*	*miR-200c*
*SOX9*	*miR-101*
*WNT4*	*miR-211*

### Expression of *CYP19A1, FST, ESR1, ESR2, SOX9*, and *WNT4 *in fetal gonads

**Figure 2:**
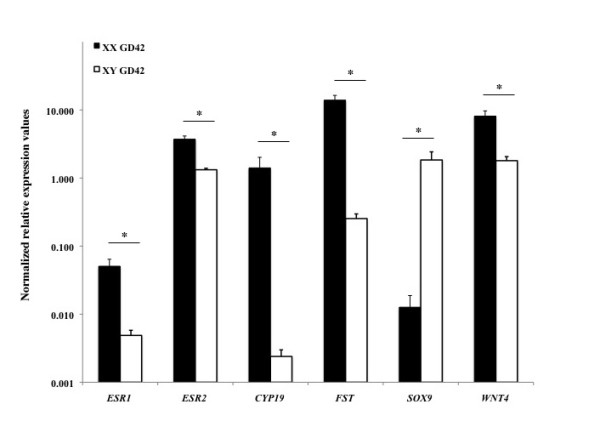
**Relative expression level of *ESR1, ESR2, CYP19A1, FST, SOX9*, and *WNT4 *in GD42 ovaries and testes according to real time PCR analysis**. *ESR1, ESR2, CYP19A1, FST*, and *WNT4 *were significantly (asterisk, P < 0.05) higher expressed in GD42 ovaries, whereas *SOX9 *was significantly (asterisk, P < 0.05) higher expressed in GD42 testes. The y-axis indicates mean 2**^-ΔCp ^**values (± SEM) according to Schmittgen and Livak, 2008 [33].

**Figure 3:**
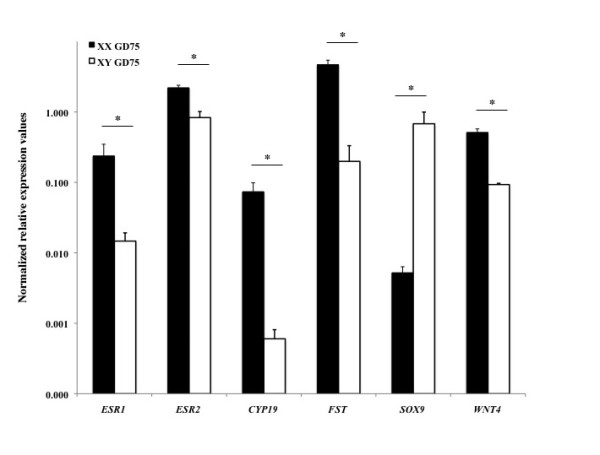
**Relative expression level of *ESR1, ESR2, CYP19, FST, SOX9*, and *WNT4 *in GD75 ovaries and testes according to real time PCR analysis**. *ESR1, ESR2, CYP19A1, FST*, and *WNT4 *were significantly (asterisk, P < 0.05) higher expressed in GD75 ovaries, whereas *SOX9 *was significantly (asterisk, P < 0.05) higher expressed in GD75 testes. The y-axis indicates mean 2**^-ΔCp ^**values (± SEM) according to Schmittgen and Livak, 2008 [33].

Expression level of a number of key genes (*SRY, CYP19A1, AMH *(anti-Mullerian hormone), *SF1*, and *WT1 *(Wilms tumor 1 homolog)) involved in fetal gonadal sex determination has been examined previously during early gonadal differentiation in sheep [37]. We confirm and extend these observations by determining the expression level of *CYP19A1, FST, ESR1, ESR2, SOX9*, and *WNT4*. Real time PCR analysis revealed that *CYP19A1, ESR1*, *ESR2*, *WNT4 *and *FST *were significant more highly expressed in ovaries compared to testes at both GD42 (Figure 2) and GD75 (Figure 3), whereas *SOX9 *was significant more highly expressed in testes compared to ovaries (Figure 2 and 3).

### MiRNA target and functional enrichment analysis

The database miRGator [36] was used to perform functional enrichment analysis of predicted miRNA targets during fetal ovarian and testicular development. For this analysis, the 10 differently expressed miRNAs exhibiting the greatest fold changes (GPR score > 0.400) between GD42 and GD75 (Table 1 and 2) were examined using the Target-Function-Expression module. During fetal ovarian and testicular development, pathways involving MAPKinase signaling, cell cycle, G-protein signaling, proteasome degradation & complex, and phospholipids as signaling intermediates are targeted more frequently by miRNAs at GD75 compared to GD42 (Table 5). Glycolysis and gluconeogenesis, and cytokines and inflammatory response pathways are targeted more frequently at both GD42 and GD75 during fetal ovarian development, whereas the WNT signaling pathway is targeted more frequently by miRNAs during fetal testicular development. Finally, regulation of ER activity is targeted by miRNAs more frequently in GD42 compared to GD75 ovaries.

**Table 5:** Functional enrichment analysis (miRGator; [36]) of predicted miRNA targets to uncover selected pathways targeted by miRNAs that are significantly more highly expressed in GD42 (downregulated) and GD75 (upregulated) ovaries and testes.

Pathway	XX		XY	
	**GD42**	**GD75**	**GD42**	**GD75**

Cell Cycle	13	20	15	20
Chromatin remodelling by hSWI/SNF ATP-dependent complexes	3	1	2	5
Cytokine Network	6	6	2	3
EGF signaling	4	3	3	2
Electron Transport chain	4	3	9	2
G-protein signaling	8	16	6	15
Gap junction proteins connexins	2	1	3	2
Glycolysis and gluconeogenesis	4	3	1	1
Growth hormone signaling	2	2	3	1
Insulin signaling	3	4	4	2
MAPKinase signaling	10	13	9	12
NGF pathway	3	2	3	
PDGF signaling pathway		4	3	3
Phospholipids as signaling intermediates		6	1	4
Proteasome degradation	1	7	1	6
Rac 1 cell motility signaling pathway	1	3	3	2
Regulation ER	5	2	1	
TGFbeta signaling pathway		2	1	3
TNFR Signaling		3		3
Transcription factor CREB and its extracellular signals	1	6	3	4
WNT signaling	5	5	7	8

### Cellular localization of miR-22 in fetal sheep gonads

**Figure 4:**
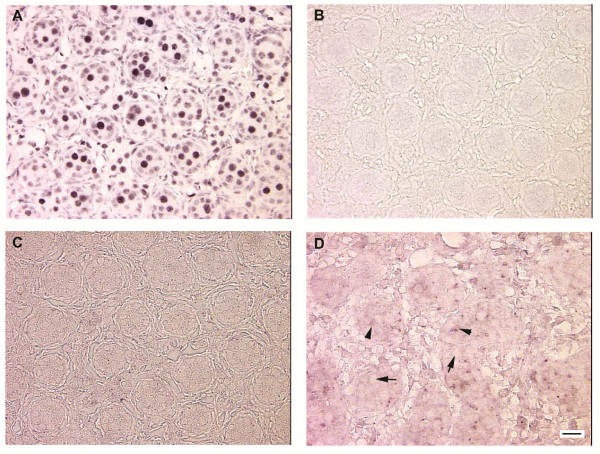
**Tissue sections of GD75 and GD90 testes**. A) GD75 testis section incubated with U6 control miRCURY LNA probe indicating positive ubiquitous staining in germ and somatic cells. B) GD75 testis section incubated with scrambled miRCURY LNA probe indicating absence of any staining. C) GD75 testis section incubated with miR-22 DIG labeled probe indicating no positive staining. D) GD90 testis section incubated with miR-22 DIG labeled probe. Arrows point to unstained gonocytes within testicular cords; arrowheads point to positive staining within testicular cords between gonocytes. All images were captured using a 40× objective, and the bar is 20 μm.

Estrogen signaling plays an important role during sheep ovarian development [27,38,39]. In situ hybridization was conducted to examine the localization of *miR-22*, predicted to target *ESR1 *in fetal sheep gonads. *MiR-22 *is down-regulated during fetal ovarian development (GPR score 0.953, ~1900 fold; Table 3) and up-regulated during testicular development (GPR score 0.500, 2.6 fold; Table 3), according to real time PCR. Using a LNA-modified probe specific to *miR-22*, in situ hybridization analysis revealed specific localization of *miR-22 *in Sertoli cells within testicular cords of GD90 testis sections, but not GD75 testis sections (Figure 4). No staining was observed within ovaries, or when a scrambled miRCURY LNA detection probe was used.

## Discussion

In mammals the activation of both fetal ovarian and testicular genetic pathways involve dynamic changes in the expression of hundred's of transcripts [10-14,40,41]. In this study we used sheep as an animal model to study the regulation of gene expression and function by miRNAs during fetal gonadal differentiation. Currently, only 4 miRNA sequences have been reported for sheep [29], however, miRNA sequences are highly conserved across species. Therefore, in order to examine miRNA expression in sheep fetal gonads, 128 miRNAs were selected that were expressed (Cp < 37; [31]) and demonstrated a single melt peak in a preliminary profiling experiment (see Additional file 1, Table S1).

Real time PCR analysis revealed that XX and XY fetal gonads contain significantly different amounts of several miRNAs. At GD42, when fetal ovaries and testes differentiate, *miR-101 *and several members of the Let7 family are preferentially expressed in testes and ovaries, respectively. One of the predicted targets of *miR-101 *is *SOX9*, which is expressed at higher relative amounts in sheep fetal testes compared to ovaries, suggesting miR-101 regulates *SOX9 *expression and/or function. Except for 137 bp (NCBI accession number AF012022), no *SOX9 *3'-UTR sequence is available for sheep. However, the 8 bp seed sequence of *miR-101 *predicted to target 3'UTR of *SOX9 *(~450 bp down stream; 5'-GUACUGU-3') is conserved (TargetScan v5.1). It is unclear how *miR-101 *regulates *SOX9 *expression or function at the transcriptional and/or post-transcriptional level during fetal testicular development, but it is possible that *miR-101 *acts to fine-tune SOX9 expression [42].

In sheep, estrogen signalling plays a role during fetal ovarian development, and formation of primordial follicles (~ GD75) may be dependent on estrogen and ESR1 signalling pathways [27,39]. CYP19A1 (aromatase) is expressed during fetal gonadal differentiation in ovaries and estrogen receptors (ESRs) are expressed as early as GD30 [37,39]. Similarly, recent studies in cows indicate that estrogen receptors and aromatase are expressed during the early stages of fetal ovarian development [43], further suggesting that estrogen signalling maybe important in fetal ovarian development in non-rodent mammalian species. MiRNA expression analysis revealed that *Let7 *and *miR-22 *are preferentially expressed in GD75 ovaries and testes, respectively. Potential targets of *Let7 *and *miR-22 *are *CYP19A1 *and *ESR1 *(*ERα*), respectively [34]. *CYP19A1 *is expressed during fetal ovarian development, whereas *Let7 *expression level is up-regulated in GD75 compared to GD42 ovaries. This suggests a possible role for *Let7 *in regulating *CYP19A1 *expression and/or function during fetal ovarian development. Similarly, *miR-22 *expression is down-regulated possibly explaining increased *ESR1 *expression level during fetal ovarian development. Importantly, Pandey and Picard [44] recently demonstrated that *miR-22 *targets and reduces *ESR1 *mRNA in breast cancer cells *in vitro*.

Contrary, in testes *CYP19A1 *and *ESR1 *expression levels are significantly lower compared to ovaries at GD42 and GD75, whereas *Let7e*, *Let7g*, and *miR-22 *expression are significantly increased in GD75 compared to GD42 testes. To examine the cellular localization of *miR-22*, LNA-modified probes and in situ hybridization was performed on GD75 testicular tissue sections. Although real time PCR indicated increased *miR-22 *expression in GD75 testes, we were unable to detect *miR-22 *using in situ hybridization at this stage. One possibility is that in situ hybridization was not sensitive enough to detect *miR-22 *at this stage. Because GD90 tissue sections were available, we explored the possibility that *miR-22 *can be detected by in situ hybridization at later stages of fetal gonadal development. *MiR-22 *localization was evident in GD90 testicular sections, and appeared to be confined within the testicular cords, localizing to the cytoplasm in Sertoli cells. Based on these results, we postulate that Sertoli cell development requires suppression of estrogen signalling during sheep testicular development involving *miR-22*.

MiRNA binding to 3'UTR target sequences occurs through complementary binding of the miRNA "seed" sequence (miRNA nucleotides 2-7). This partially explains how miRNAs potentially can regulate expression and/or function of more than one mRNA or whole signalling networks and complex biological processes. Using a bioinformatics approach we explored the possibility that the different biological processes or pathways underlying fetal ovarian and testicular development are regulated by miRNAs. This analysis revealed that estrogen signalling (regulation of ESR and modulation ESR activity) is targeted less frequently by miRNAs as fetal ovarian development proceeds. For example, during ovarian development (comparing GD42 to GD75 ovaries) the number of miRNAs targeting ESR function decreases, suggesting that estrogen function is necessary for proper ovarian and follicle development at GD75.

Another example of signalling pathways targeted by miRNAs in fetal sheep gonads is G-protein signalling (includes G-protein signalling, G_13 _signalling pathway, signalling pathway from G protein families). Both in fetal ovaries and testes, the number of miRNAs targeting G-protein signalling increases during gonadal development, suggesting that miRNAs potentially are involved in regulating G-protein signalling mediated functions during the latter stages of fetal gonadal development. G-protein signalling encompassing many different protein families and different cell signalling pathways is known to regulate reproduction at the hypothalamic, pituitary, and gonadal level [45]. During ovarian development G-protein-coupled receptor (GPR) 30, which has high affinity for estradiol, is involved in primordial follicle formation in hamster ovaries [46]. A predicted binding site for *miR-130a *(significant more highly expressed in GD75 compared to GD42 ovaries) is present within the 3'UTR of (human) *GPR30*.

Although this bioinformatics approach suggests the potential regulation of signaling pathways (or other biological processes) by miRNAs, future studies demonstrating direct interaction of selected miRNAs with their target sequences will need to be provided.

## Conclusions

In summary, data presented in this study indicate that miRNAs are present during fetal gonadal development and differentiation in sheep. Based on the correlations observed between miRNA expression and their predicted targets, we postulate that miRNAs are important regulators of gene expression and function during fetal gonadal development. Similar to what has been proposed for the cow [47], the sheep is a useful model to study gonadal development and differentiation in mammals. For example, estrogen signalling appears to play a role in human, cow, and sheep fetal ovarian development [38,39,43,48]. Based on our results, we further suggest that *Let7 *and *miR-22 *regulate estrogen signalling during fetal sheep gonadal development, and *miR-22 *may be necessary for suppressing the estrogen-signalling pathway during fetal testicular development. Finally, bioinformatic analysis revealed several pathways that are possibly regulated by miRNAs during fetal ovarian as well as testicular development.

## Competing interests

The authors declare that they have no competing interests.

## Authors' contributions

KJT and JCS performed the experiments, and DNRV helped with the immunohistochemistry analyses. PS, RVA, DNRV, and QAW provided reagents and materials. GJB designed the experiments, supervised the study, and wrote the manuscript. All authors read and approved the final manuscript.

## Supplementary Material

Additional file 1 **Supplemental Table S1: The 128 mature miRNAs examined in this study**. The mature miRNA sequence was used as the forward primer sequence in the real time PCR analysis. Click here for file

Additional file 2 **Supplemental Table S2: Gene specific primer sequences used to examine mRNAs by real time PCR**. Click here for file
