# Supplementary material for: Expression of miRNAs in ovine fetal gonads: potential role in gonadal differentiation
Source: Reprod Biol Endocrinol. 2011 Jan 11;9:2. doi: 10.1186/1477-7827-9-2 (PMC3027096; doi:10.1186/1477-7827-9-2)
Supplement: Additional file 1 — Supplemental Table S1: The 128 mature miRNAs examined in this study. The mature miRNA sequence was used as the forward primer sequence in the real time PCR analysis. [file 1477-7827-9-2-S1.DOC]

| mature miRNA ID | microRNA Mature Sequence |
| --- | --- |
| let-7a | UGAGGUAGUAGGUUGUAUAGUU |
| let-7c | UGAGGUAGUAGGUUGUAUGGUU |
| let-7d | AGAGGUAGUAGGUUGCAUAGU |
| let-7e | UGAGGUAGGAGGUUGUAUAGU |
| let-7g | UGAGGUAGUAGUUUGUACAGU |
| mir-100 | AACCCGUAGAUCCGAACUUGUG |
| mir-101 | UACAGUACUGUGAUAACUGAAG |
| mir-103 | AGCAGCAUUGUACAGGGCUAUGA |
| mir-107 | AGCAGCAUUGUACAGGGCUAUCA |
| mir-10a | UACCCUGUAGAUCCGAAUUUGUG |
| mir-10b | UACCCUGUAGAACCGAAUUUGU |
| mir-125a | UCCCUGAGACCCUUUAACCUGUG |
| mir-125b | UCCCUGAGACCCUAACUUGUGA |
| mir-127 | UCGGAUCCGUCUGAGCUUGGCU |
| mir-130a | CAGUGCAAUGUUAAAAGGGCAU |
| mir-132 | UAACAGUCUACAGCCAUGGUCG |
| mir-134 | UGUGACUGGUUGACCAGAGGG |
| mir-135a | UAUGGCUUUUUAUUCCUAUGUGA |
| mir-138 | AGCUGGUGUUGUGAAUC |
| mir-142-3p | CAUAAAGUAGAAAGCACUAC |
| mir-142-5p | UGUAGUGUUUCCUACUUUAUGGA |
| mir-143 | UGAGAUGAAGCACUGUAGCUCA |
| mir-146b | UGAGAACUGAAUUCCAUAGGCU |
| mir-148a | UCAGUGCACUACAGAACUUUGU |
| mir-148b | UCAGUGCAUCACAGAACUUUGU |
| mir-149 | UCUGGCUCCGUGUCUUCACUCC |
| mir-150 | UCUCCCAACCCUUGUACCAGUG |
| mir-152 | UCAGUGCAUGACAGAACUUGGG |
| mir-15a | UAGCAGCACAUAAUGGUUUGUG |
| mir-15b | UAGCAGCACAUCAUGGUUUACA |
| mir-16 | UAGCAGCACGUAAAUAUUGGCG |
| mir-17-3p | CAAAGUGCUUACAGUGCAGGUAGU |
| mir-17-5p | ACUGCAGUGAAGGCACUUGU |
| mir-181b | AACAUUCAUUGCUGUCGGUGGG |
| mir-181c | AACAUUCAACCUGUCGGUGAGU |
| mir-182 | UUUGGCAAUGGUAGAACUCACA |
| mir-183 | UAUGGCACUGGUAGAAUUCACUG |
| mir-191 | CAACGGAAUCCCAAAAGCAGCU |
| mir-192 | CUGACCUAUGAAUUGACAGCC |
| mir-193a | AACUGGCCUACAAAGUCCCAG |
| mir-194 | UGUAACAGCAACUCCAUGUGGA |
| mir-195 | UAGCAGCACAGAAAUAUUGGC |
| mir-196a | UAGGUAGUUUCAUGUUGUUGG |
| mir-196b | UAGGUAGUUUCCUGUUGUUGG |
| mir-199a | CCCAGUGUUCAGACUACCUGUUC |
| mir-199b | CCCAGUGUUUAGACUAUCUGUUC |
| mir-19a | UGUGCAAAUCUAUGCAAAACUGA |
| mir-19b | UGUGCAAAUCCAUGCAAAACUGA |
| mir-200b | UAAUACUGCCUGGUAAUGAUGAC |
| mir-200c | UAAUACUGCCGGGUAAUGAUGG |
| mir-204 | UUCCCUUUGUCAUCCUAUGCCU |
| mir-206 | UGGAAUGUAAGGAAGUGUGUGG |
| mir-20a | UAAAGUGCUUAUAGUGCAGGUAG |
| mir-20b | CAAAGUGCUCAUAGUGCAGGUAG |
| mir-21 | UAGCUUAUCAGACUGAUGUUGA |
| mir-210 | CUGUGCGUGUGACAGCGGCUGA |
| mir-211 | UUCCCUUUGUCAUCCUUCGCCU |
| mir-212 | UAACAGUCUCCAGUCACGGCC |
| mir-214 | ACAGCAGGCACAGACAGGCAG |
| mir-216 | UAAUCUCAGCUGGCAACUGUG |
| mir-219 | UGAUUGUCCAAACGCAAUUCU |
| mir-22 | AAGCUGCCAGUUGAAGAACUGU |
| mir-221 | AGCUACAUUGUCUGCUGGGUUUC |
| mir-222 | AGCUACAUCUGGCUACUGGGUCUC |
| mir-223 | UGUCAGUUUGUCAAAUACCCC |
| mir-23a | AUCACAUUGCCAGGGAUUUCC |
| mir-23b | AUCACAUUGCCAGGGAUUACC |
| mir-24 | UGGCUCAGUUCAGCAGGAACAG |
| mir-25 | CAUUGCACUUGUCUCGGUCUGA |
| mir-26a | UUCAAGUAAUCCAGGAUAGGC |
| mir-27a | UUCACAGUGGCUAAGUUCCGC |
| mir-27b | UUCACAGUGGCUAAGUUCUGC |
| mir-28 | AAGGAGCUCACAGUCUAUUGAG |
| mir-296 | AGGGCCCCCCCUCAAUCCUGU |
| mir-301 | CUUUCAGUCGGAUGUUUGCAGC |
| mir-302d | UGUAAACAUCCUCGACUGGAAG |
| mir-30a-3p | CAGUGCAAUAGUAUUGUCAAAGC |
| mir-30a-5p | UAAGUGCUUCCAUGUUUGAGUGU |
| mir-30b | UGUAAACAUCCUACACUCAGCU |
| mir-30c | UGUAAACAUCCUACACUCUCAGC |
| mir-30d | UGUAAACAUCCCCGACUGGAAG |
| mir-30e-5p | UGUAAACAUCCUUGACUGGA |
| mir-320 | AAAAGCUGGGUUGAGAGGGCGAA |
| mir-324-5p | CGCAUCCCCUAGGGCAUUGGUGU |
| mir-328 | CUGGCCCUCUCUGCCCUUCCGU |
| mir-33 | GUGCAUUGUAGUUGCAUUG |
| mir-331 | GCCCCUGGGCCUAUCCUAGAA |
| mir-335 | UCAAGAGCAAUAACGAAAAAUGU |
| mir-339 | UCCCUGUCCUCCAGGAGCUCA |
| mir-340 | UCCGUCUCAGUUACUUUAUAGCC |
| mir-342 | UCUCACACAGAAAUCGCACCCGUC |
| mir-34c | AGGCAGUGUAGUUAGCUGAUUGC |
| mir-361 | UUAUCAGAAUCUCCAGGGGUAC |
| mir-362 | AAUCCUUGGAACCUAGGUGUGAGU |
| mir-369-5p | AGAUCGACCGUGUUAUAUUCGC |
| mir-376a | AUCAUAGAGGAAAAUCCACGU |
| mir-377 | AUCACACAAAGGCAACUUUUGU |
| mir-379 | UGGUAGACUAUGGAACGUA |
| mir-382 | GAAGUUGUUCGUGGUGGAUUCG |
| mir-409-5p | AGGUUACCCGAGCAACUUUGCA |
| mir-410 | AAUAUAACACAGAUGGCCUGU |
| mir-411 | UAGUAGACCGUAUAGCGUACG |
| mir-421 | AUCAACAGACAUUAAUUGGGCGC |
| mir-423 | AGCUCGGUCUGAGGCCCCUCAG |
| mir-425-5p | AAUGACACGAUCACUCCCGUUGA |
| mir-431 | UGUCUUGCAGGCCGUCAUGCA |
| mir-433 | AUCAUGAUGGGCUCCUCGGUGU |
| mir-451 | AAACCGUUACCAUUACUGAGUUU |
| mir-455 | UAUGUGCCUUUGGACUACAUCG |
| mir-484 | UCAGGCUCAGUCCCCUCCCGAU |
| mir-485-5p | AGAGGCUGGCCGUGAUGAAUUC |
| mir-486 | UCCUGUACUGAGCUGCCCCGAG |
| mir-497 | CAGCAGCACACUGUGGUUUGU |
| mir-539 | GGAGAAAUUAUCCUUGGUGUGU |
| mir-574 | CACGCUCAUGCACACACCCAC |
| mir-598 | UACGUCAUCGUUGUCAUCGUCA |
| mir-615 | UCCGAGCCUGGGUCUCCCUCU |
| mir-652 | AAUGGCGCCACUAGGGUUGUGCA |
| mir-668 | UGUCACUCGGCUCGGCCCACUAC |
| mir-675 | UGGUGCGGAGAGGGCCCACAGUG |
| mir-7 | UGGAAGACUAGUGAUUUUGUUG |
| mir-758 | UUUGUGACCUGGUCCACUAACC |
| mir-801 | GAUUGCUCUGCGUGCGGAAUCGAC |
| mir-92 | UAUUGCACUUGUCCCGGCCUG |
| mir-99a | AACCCGUAGAUCCGAUCUUGUG |
| mir-99b | CACCCGUAGAACCGACCUUGCG |
| U6 | CGCAAGGAUGACACGCAAAUUC |
| RNU43 | CUUAUUGACGGGCGGACAGAAAC |
