# Supplementary material for: Expression of miRNAs in ovine fetal gonads: potential role in gonadal differentiation
Source: Reprod Biol Endocrinol. 2011 Jan 11;9:2. doi: 10.1186/1477-7827-9-2 (PMC3027096; doi:10.1186/1477-7827-9-2)
Supplement: Additional file 2 — Supplemental Table S2: Gene specific primer sequences used to examine mRNAs by real time PCR. [file 1477-7827-9-2-S2.DOC]

|  | Forward Primer | Reverse Primer |
| --- | --- | --- |
| Gene Symbol | 5' to 3' | 5' to 3' |
| *CYP19A1* | GTTGTGCCTATTGCCAGCAT | AACCTGCAGTGGGAAATGAG |
| *ESR1* | CGCGTCCTGGACAAGATCAC | TGCTCCATGCCTTTGTTGCT |
| *ESR2* | CTGTCGACTGCGGAAGTGCT | ATGGGTGCACCGTTCCTCTT |
| *FST* | CAACACGCTGTTCAAGTGGATG | GCGGGGTTTGTTCTTCTTGTTC |
| *SOX9* | GCAAGCTCTGGAGGCTGCT | CTCCGCGGCTGGTACTTGT |
| *WNT4* | AGGTGGTGACGCAAGGGACT | GTCCGGTCACAGCCACACTT |
|  |  |  |
| *RN18S* | GAGGCCCTGTAATTGGAATGAG | GCAGCAACTTTAATATACGCTATTGG |
| *GAPDH* | GATTGTCAGCAATGCCTCCT | GGTCATAAGTCCCTCCACGA |
